# Lipofuscin accumulation in aging and neurodegeneration: a potential “timebomb” overlooked in Alzheimer’s disease

**DOI:** 10.1186/s40035-025-00529-x

**Published:** 2025-12-12

**Authors:** Godfried Dougnon, Hideaki Matsui

**Affiliations:** Department of Neuroscience of Disease, Brain Research Institute, Niigata University, Niigata, 951-8585 Japan

**Keywords:** Lipofuscin, Neurodegeneration, Alzheimer’s disease, Amyloid beta (Aβ), Aging, Oxidative stress, Inflammation, Lysosomal dysfunction, Autofluorescence, Reactive oxygen species

## Abstract

Lipofuscin, a marker of aging, is the accumulation of autofluorescent granules within microglia and postmitotic cells such as neurons. Lipofuscin has traditionally been regarded as an inert byproduct of cellular degradation. However, recent findings suggest that lipofuscin may play a role in modulating age-related neurodegenerative processes, and several questions remain unanswered. For instance, why do lipofuscin granules accumulate preferentially in aged neurons and microglia? What happens to these pigments upon neuronal demise? Particularly in neurodegenerative diseases like Alzheimer’s disease (AD), why does amyloid β (Aβ) deposition usually begin in late adulthood or during aging? Why do lipofuscin and amyloid plaques appear preferentially in grey matter and rarely in white matter? In this review, we argue that lipofuscin should be revisited not as a simple biomarker of aging, but as a potential modulator of neurodegenerative diseases. We synthesize emerging evidence linking lipofuscin to lysosomal dysfunction, oxidative stress, lipid peroxidation and disease onset—mechanisms critically implicated in neurodegeneration. We also explore the potential interactions of lipofuscin with Aβ and their spatial location, and summarize evidence showing that lipofuscin may influence disease progression via feedback loops affecting cellular clearance and inflammation. Finally, we propose future research directions toward better understanding of the mechanisms of lipofuscin accumulation and improved lysosomal waste clearance in aging.

## Introduction

Alzheimer's disease (AD) is a major neurodegenerative disease defined by progressive cognitive decline and neuropathological traits, such as extracellular amyloid-β (Aβ) plaques (Table 1) and intracellular neurofibrillary tangles (NFTs) composed of hyperphosphorylated tau protein [1]. The amyloid hypothesis of AD suggests that the accumulation of Aβ peptides initiates a cascade of events leading to neurodegeneration [2–4]. This hypothesis is centered on the idea that the accumulation of Aβ peptides derived from amyloid precursor protein (APP) plays a major role in disease development. APP is particularly renowned for its pathogenic cleavage by β- and γ-secretases leading to the production of Aβ peptides, which can aggregate and form amyloid plaques [5].

**Table 1:** Important terms and definitions related to this review

Terms	Definitions
Amyloid β (Aβ)	A 38–43 amino acid peptide implicated in Alzheimer’s disease, typically viewed as a cleavage product of APP. In this review, we summarize articles demonstrating that it is a secondary product of neuronal decay and lysosomal egress
Amyloidogenesis	The formation of amyloid aggregates; a consequence of neuronal demise potentiated by released lipofuscin and toxic environmental conditions
Lipofuscin	An autofluorescent pigment comprised of oxidized proteins, lipids, and metals that accumulate in the lysosomes of microglia and postmitotic cells, such as neurons. Here, we describe it as a potential initiator of extracellular amyloid pathology upon lysosomal dysfunction and neuronal death
Lysosomal exocytosis	The process by which lysosomes fuse with the plasma membrane and proceed to the expulsion of their contents outside the cell. This process is crucial for clearing waste, repairing membranes, and maintaining cellular homeostasis—especially under stress
Lysosomal failure	A breakdown in autophagic and lysosomal degradation pathways, ultimately leading to intracellular waste buildup and potential release upon cell death
Senolysis	Pharmacologic clearance of senescent or dysfunctional cells; here, it is proposed as a strategy to prevent inflammatory and proteopathic aftermath of cell death

However, the weak correlation between plaque burden and cognitive impairment has prompted investigations into additional factors contributing to AD pathology [6, 7]. Moreover, the AD hypothesis does not explain what triggers the accumulation of Aβ in the brain. Considering that Aβ symptomatology begins later in life and the fact that aging is accompanied by the accumulation of waste material [8, 9], it is highly possible that other factors related to aging contribute to disease onset.

Lipofuscin (Table 1) is an autofluorescent, electron-dense pigment that accumulates with age in microglia and postmitotic cells, such as neurons [10]. Composed primarily of oxidized proteins and lipids, lipofuscin is considered as a marker of aging and is indicative of oxidative stress and impaired lysosomal degradation [11, 12]. While lipofuscin is traditionally viewed as a benign byproduct of aging, recent studies suggest that its accumulation may exert deleterious effects on cellular function and viability [11, 13–15]. Lipofuscin is most commonly identified by its intrinsic autofluorescence, particularly in the red spectrum (570–640 nm). Histochemically, Sudan Black B [16, 17] and classical reactions such as the Schmorl’s method [18, 19], together with ultrastructural confirmation by electron microscopy [20, 21], remain the principal approaches for identification. By contrast, there are no universally accepted molecular markers for lipofuscin. However, studies investigating lipofuscin have remained limited.

In this review, we examine the potential interplay between lipofuscin accumulation, lysosomal dysfunction, lipid peroxidation and Aβ pathology in AD. We explore how lipofuscin may influence Aβ aggregation, clearance, and toxicity and propose mechanisms by which lipofuscin modulates AD progression. Importantly, we summarize evidence demonstrating that lipofuscin is released extracellularly upon neuronal death, thus preparing a highly oxidized environment that results in toxicity and a cascade of events leading to plaque formation and Aβ pathology.

## Lipofuscin accumulation and lysosomal dysfunction

Lipofuscin is traditionally characterized by two major approaches: strong autofluorescence under light microscopy, and distinct ultrastructural features under electron microscopy. In this review, unless otherwise specified, the evidence discussed refers to light-microscopic identification through autofluorescence, which is the most widely used method in histological and imaging analyses of lipofuscin. Lipofuscin accumulates within lysosomes as a result of incomplete degradation of cellular components, and this phenomenon is considered part of normal aging [10, 14]. Additionally, lipofuscin, which is rich in redox-active metals such as iron and copper, contributes to the production of reactive oxygen species (ROS) through Fenton reactions [22, 23]. In living cells, efficient lysosomal degradation is crucial for the clearance of unwanted materials. Impaired lysosomal function due to lipofuscin build-up could therefore prevent their degradation, promoting aggregation and a toxic environment [24, 25]. Lipofuscin has been found in microglial lysosomes even in young brains [26], suggesting that its accumulation is progressive and may reflect early lysosomal stress rather than just aging. This early accumulation renders microglia more susceptible to functional impairments, which eventually impairs their capacity to clear extracellular debris and thus exacerbates plaque formation [27]. Additionally, due to its composition, lipofuscin creates an oxidative environment that can damage lysosomal membranes, leading to the leakage of hydrolytic enzymes into the cytosol and enhancing cellular damage [22, 23].

Recent studies have demonstrated important involvement of lysosomal dysfunction in the pathogenesis of neurodegenerative diseases. For instance, neuronal ceroid lipofuscinosis (NCL) has been linked to mutations of genes related to lysosomal function, including *GRN* (granulin), *CTSD* (cathepsin D) and *CLN5* (ceroid lipofuscinosis neuronal protein 5) [28–32]. Interestingly, many of these genes are also risk factors of AD and their mutations can lead to impaired lysosomal activity, resulting in accumulation of undegraded substrates such as lipofuscin. Moreover, the lysosomal storage of lipofuscin can disrupt calcium homeostasis and mitochondrial function, and aggravate neuronal dysfunction, causing death [33, 34]. Progranulin (PGRN), which is a secreted protein encoded by *GRN* and produced by myeloid cells and neurons, plays an immunoregulatory role and contributes to the regulation of lysosomal function. It also supports neuronal survival and rescues inflammation [30, 35]. In particular, *GRN* loss-of-function mutations cause NCL and frontotemporal dementia (FTD)-GRN [36, 37]. Similarly, as described in patients with Kufs disease, pathological lipofuscinosis and intracellular structures resembling granular osmiophilic deposits are observed in animal models, and are engulfed by microglia [38, 39]. Notably, Kufs disease is the major adult form of NCL, and its diagnosis is based on the evidence of specific storage material; however, distinction of this material from lipofuscin is challenging [39]. Kufs disease often develops in adolescence or adulthood, with symptoms appearing in the late 20’s to early 50’s. A hallmark of this disease is progressive dementia, which is associated with movement disorders such as ataxia (loss of coordination), myoclonus, and seizures [38, 40]. This evidence suggests a role for lipofuscin accumulation in preparing an environment for neurodegeneration.

In addition, variants in *SORL1* (sortilin related receptor 1) affect trafficking of APP and lysosomal function, and *CTSD* encodes cathepsin D, a major lysosomal protease [31, 41, 42]. Beyond these, the strongest genetic risk factor for sporadic AD, *APOE4*, is also linked to lysosomal pathway abnormalities, including impaired lipid transport and disruption of endosomal trafficking that secondarily impacts lysosomal function [43]. Mutations in *CLN5*, which can cause one of the 13 forms of NCL and are also linked to AD, are reported to cause accumulation of autofluorescent material and impede lysosomal function [32, 44, 45].

Beyond risk alleles, autosomal-dominant mutations in *APP*, *PSEN1* (presenilin 1), and *PSEN2* (presenilin 2), which are causative of early-onset familial AD, have been demonstrated to directly compromise lysosomal function. These mutations, by affecting γ-secretase activity and APP metabolism, lead to abnormal lysosomal acidification, lower cathepsin activity, and impairments in autophagic clearance. For instance, recent work has shown that APP processing and presenilin function converge on lysosomal homeostasis, suggesting that lysosomal dysfunction may be a central mechanism linking familial and sporadic AD pathogenesis [46–48].

Together, these findings broaden the genetic landscape connecting AD to lysosomal biology. The converging evidence from risk alleles (*APOE4*, *SORL1*, *GRN*, *CTSD*, *CLN5*) and causative mutations (*APP*, *PSEN1*, *PSEN2*) underscores that lysosomal dysfunction is not a secondary consequence of pathology, but rather a genetically supported and likely initiating factor in disease pathogenesis. Furthermore, as lipofuscin accumulates over time, it progressively impairs lysosomal activity, creating a perpetuating cycle of dysfunction, a phenomenon previously called "garbage catastrophe" [49]. This interplay between lipofuscin accumulation and lysosomal dysfunction creates a noxious cycle that exacerbates neurodegeneration.

## Spatial relationship between lipofuscin and Aβ

The spatial distribution of lipofuscin and Aβ in aging and AD is a subject receiving increasing interest. Understanding their colocalization—or lack thereof—could reveal important mechanistic clues about their interplay. D’Andrea et al. have found spatial segregation between lipofuscin and Aβ [50]. They used a novel combined immunohistochemical:histochemical staining protocol for detection, and mentioned 4 different antibodies from companies, namely Quality Controlled Biochemicals-Biosource, Pharmingen, Oncogene, and Alpha Diagnostics International; however, data of the different antibodies were not shown for comparative analysis [50]. On the contrary, Aβ and lipofuscin are often reported to be both intracellular and extracellular [51–53]. Intracellular Aβ has been reported exclusively in patients with Osaka mutation of *APP* gene, another cause of dementia [54]. Analysis of specimens carrying the Osaka mutation could reveal a similar composition to that of lipofuscin. The spatial distribution pattern of lipofuscin implies that it might share with Aβ some upstream mechanisms of production or clearance failure.

Electron microscopy studies have revealed that lipofuscin granules are in close proximity to autophagosomes and multilamellar bodies containing Aβ_42_ peptides [25, 55]. These observations suggest that lipofuscin accumulation may disrupt the autophagic–lysosomal trafficking, facilitating Aβ aggregation by preventing its degradation. Nevertheless, new research has indicated that aging and AD can induce autophagy dysfunction, facilitating lipofuscin production and accumulation inside neurons [47]. Brunk and Terman have described how the accumulation of indigestible materials such as lipofuscin can contribute to functional reduction of the cellular degradative capacity [56, 57].

Interestingly, the anatomical distribution of lipofuscin accumulation also significantly overlaps with the brain regions most affected in AD. Giaccone et al. [27] first proposed a hypothesis that neuronal death and the subsequent release of lipofuscin granules into the extracellular milieu may provide a niche for Aβ aggregation and plaque formation. This theory is supported by recent findings showing that lipofuscin itself can be immunoreactive with antibodies raised against Aβ [58–61]. Indeed, early immunohistochemical studies demonstrated that neuronal lipofuscin granules, as well as ceroid pigments in lysosomal storage disorders, are recognized by monoclonal antibodies for Aβ. For instance, in Batten disease (or NCL), ceroid-lipofuscin storage pigment exhibits strong immunoreactivity to Aβ antibodies, raising the possibility of structural or epitope similarities between Aβ aggregates and lipofuscin components [60]. Bancher et al. [59] further extended this finding in human AD brains, demonstrating that lipofuscin within neurons could be labeled by anti-Aβ antibodies in addition to extracellular amyloid plaques [58]. Another group further confirmed that a monoclonal antibody to the Aβ peptide labeled both amyloid deposits and intracellular pigments across multiple tissues. All these lines of evidence suggest shared antigenic determinants. It should be noted that these results were largely based on immunohistochemistry using monoclonal antibodies such as 4G8 and 6E10, which recognize epitopes within the Aβ sequence but can also bind to *APP*-derived fragments. Consequently, it remains unresolved whether the observed immunoreactivity reflects Aβ peptide, APP-CTFβ, or cross-reactive epitopes that are present in oxidized proteins and lipids comprising lipofuscin.

**Fig. 1:**
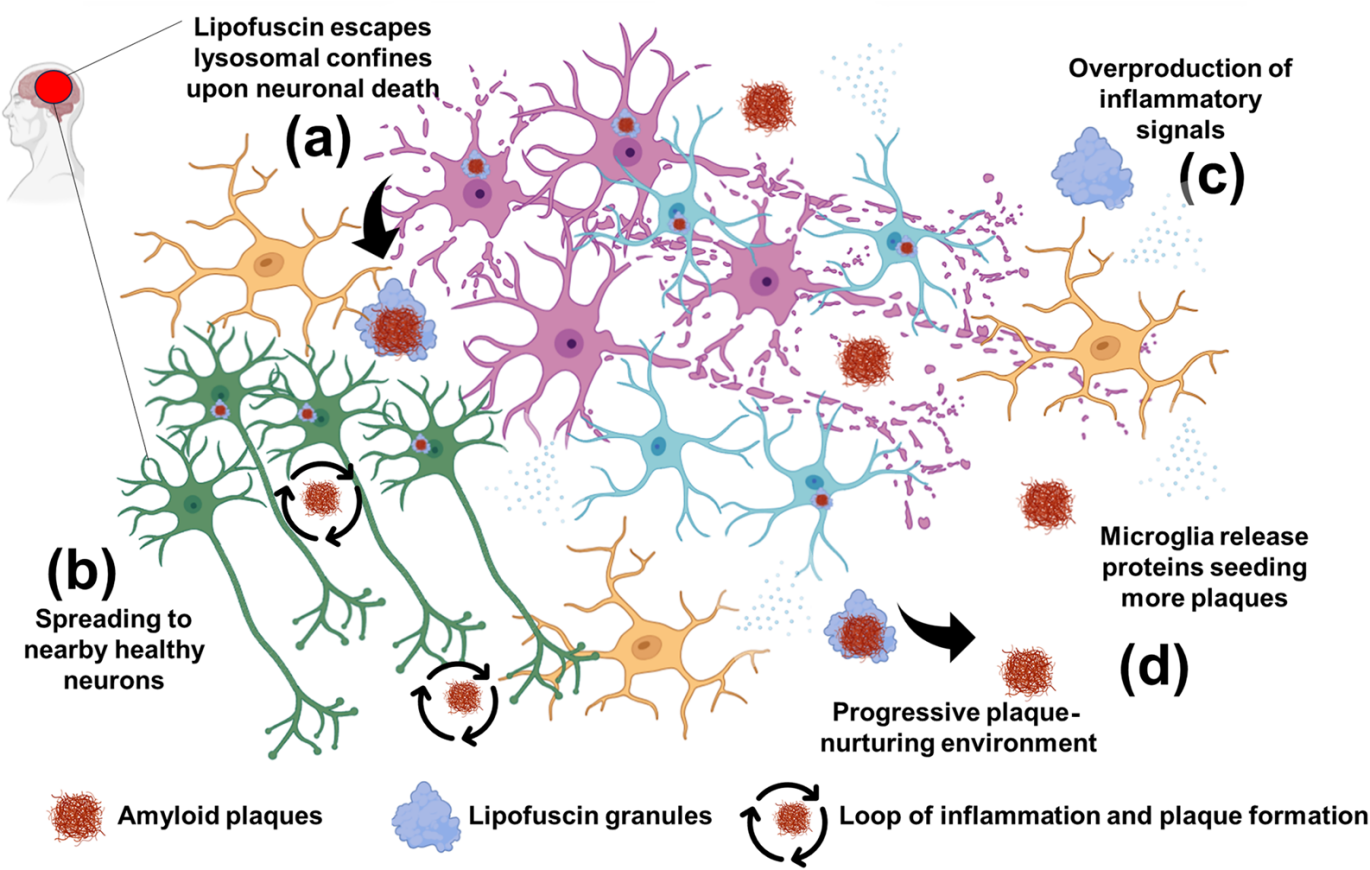
Lipofuscin-induced inflammation and plaque propagation in neurodegeneration. Upon neuronal death, lipofuscin with intracellular Aβ escapes from the lysosome and is released into the extracellular environment (**a**), triggering microglial activation and overproduction of inflammatory signals. The activated microglia further secrete proteins that seed pathological plaques, creating an even more toxic microenvironment, facilitating the spread of neurodegenerative pathology to nearby healthy neurons (**b** and **c**). This loop of inflammation, plaque formation, and neuronal damage places lipofuscin granules as potential “timebombs” and causes the progression of neurodegenerative diseases such as AD (**d**)

Difficulties in discriminating between lipofuscin fragments and intracellular Aβ have also been reported [62]. For instance, lipofuscin aggregates were shown to be widely distributed throughout the sections of prefrontal cortex from an AD patient (section treated with 70% formic acid) and particularly demonstrated sporadic colocalization with extracellular Aβ plaques. This underscores the need for rigorous controls including spectral analysis, quenching of autofluorescence, and use of multiple antibody clones. A further nuance that is interesting is the spatial context of Aβ immunoreactivity. Early studies often described Aβ-like staining within neurons of aged animals, and this intraneuronal labeling could be interpreted to reflect APP processing intermediates sequestered in lysosomal compartments. Upon neuronal death, these intracellular deposits are released into the extracellular space, and may serve as seeds or scaffolds for the formation of extracellular Aβ plaques (Fig. 1). Thus, the cross-reactivity of Aβ antibodies with lipofuscin may represent both (i) intraneuronal recognition of pigment-associated APP or Aβ-related epitopes and (ii) extracellular persistence of lipofuscin granules that colocalize with or promote plaque formation. Our recent work [63] builds upon these observations by showing that while APP partly colocalized with lipofuscin autofluorescence, the intracellular signals that were recognized by an antibody directed against Aβ (BC05, which recognizes amino acids 1–16 of the Aβ peptide) almost disappeared following fluorescence quenching treatment with TrueBlack. However, because TrueBlack’s composition is proprietary to the manufacturer, it is possible that the intracellular signal was genuine Aβ that was not strong enough to resist TrueBlack treatment. Taken together, these data highlight the need for careful methodological distinctions between lipofuscin autofluorescence, Aβ immunoreactivity, and APP-derived fragments, while supporting the broader concept that lipofuscin release into the extracellular space may play an underappreciated role in seeding or amplifying amyloid pathology in AD.

Recent studies suggest that lipofuscin, long considered primarily as an intracellular age pigment, may also be present in the extracellular space in the context of AD. Notably, sporadic colocalization of lipofuscin granules with extracellular Aβ plaques has been documented [62], providing preliminary support for the hypothesis that pigment release from degenerating neurons could contribute to plaque formation. Interestingly, Ahmed et al. [64] found a population of microglia in old *Grn*^−/−^ mice that exhibited lipofuscin accumulation apparently independent from the neuronal lipofuscin. These phagocytic microglia were absent at younger ages, which suggests that they appear from microglia trying to phagocyte lipofuscin-burdened neurons. The researchers also reported a particular type of lipofuscin material that was extracellular, had a perivascular distribution, and was negative for ubiquitin staining but positive for Luxol Fast Blue and Periodic Acid-Schiff staining, in regions like the thalamus, CA2 and CA3 of hippocampus, and the brainstem [64]. Furthermore, Cataldo et al. [65] reported lipofuscin aggregates in the extracellular space following cell lysis, and these aggregates were often associated with Aβ deposits. They noted that these aggregates were mainly from degenerating neurons and their processes containing high concentrations of acid hydrolase. Additionally, another study using energy dispersive X-ray spectrometry with scanning electron microscopy of purified lipofuscin granules from autopsied brains with AD, and fluorescence microscopy of Bodian-stained AD brains, revealed that the aluminum detected in senile plaques and NFTs originates from lipofuscin, which itself is concentrated in the plaques and tangles [66]. Braak and Braak have reported that neurons burdened by lipofuscin accumulation could easily develop AD related changes [67, 68]. Of note, lipofuscin pigments have been identified in the extracellular space of aged horse brains [69]. Nevertheless, while intraneuronal lipofuscin accumulation is well established, systematic quantification of extracellular lipofuscin and its spatial relationship to plaques remains scarce.

While it is plausible that lipofuscin shares antigenic determinants with Aβ, more evidence points toward that Aβ is actually degraded in lysosomes, where it accumulates with lipofuscin in aging and neurodegeneration [47, 70–73]. Therefore, the interpretation that Aβ has antigenic relationship with lipofuscin might be misleading. Aβ and amyloidogenic APP metabolites are normally trafficked to lysosomes for degradation, and as lysosomal efficiency declines with aging and AD, the substrates accumulate within lysosomes and lipofuscin-related organelles [47, 70]. Therefore, the Aβ immunoreactivity within lipofuscin granules can be caused by the trapping of incompletely degraded Aβ in these organelles, rather than that lipofuscin itself contains amyloid epitopes. It is also essential to highlight that in AD, a typical observation is the buildup of enlarged, lysosome-related organelles containing lipofuscin-like pigments that also sequester undegraded Aβ.

This distinction refines our understanding of the relationship between lipofuscin and amyloid pathology. It emphasizes that lysosomal degradation failure is the initiating event, with lipofuscin granule accumulation representing both a marker and a mechanism of impaired clearance. Aβ becomes trapped in this process, further amplifying lysosomal stress and contributing to the transition from intracellular accumulation to extracellular amyloid deposition.

Based on the fact that circadian clock and autophagy are involved in aging [74, 75], Börner et al. [74] have investigated the entorhinal cortex, hippocampus, and neocortex of *Per1*^−/−^ aged mice. These areas are characterized by increased neuronal activity, more metabolic activity, and susceptibility to proteostasis failure; interestingly, they also exhibit accumulation of lipofuscin and Aβ [74]. Moreover, lipofuscin and Aβ are present in less amount in the white matter, and exist predominantly in the grey matter [10, 76, 77]. Recent studies have found elevated levels of lipofuscin in the grey matter of postmortem AD brains compared to controls [78]. This anatomical concordance warrants further validation via spatially resolved analyses using techniques such as multiplexed imaging or spatial transcriptomics. Thus, the spatial co-occurrence of lipofuscin and Aβ supports the hypothesis of potential functional interplay between them, which at least is mediated by impairment of their degradation, trafficking, and oxidative balance.

## Amyloidogenesis as a byproduct of intracellular lipofuscin release upon neuronal death

There has been mounting evidence challenging the view that Aβ plaque deposition is the earliest pathogenic event in AD. Instead, converging studies highlight lysosomal dysfunction as a pivotal process that may precede and even drive Aβ accumulation. Nixon emphasized that disruption of the autophagic-lysosomal system occurs prior to extracellular plaque deposition, leading to intraneuronal accumulation of Aβ and other aggregates. Therefore, amyloid plaques may represent a downstream manifestation of earlier endolysosomal defects rather than being an initiating cause [47]. This perspective reframes amyloid accumulation as a consequence of impaired degradation pathways rather than aberrant overproduction alone. Studies by Cataldo et al. have also provided some of the earliest evidence of lysosomal storage pathology in AD neurons, showing that endosomal and lysosomal compartments are markedly enlarged and dysfunctional even in the prodromal phase of the disease [79, 80]. Glabe’s review work complements these findings by demonstrating that intracellular accumulation of soluble Aβ oligomers, potentiated by defective clearance mechanisms, can trigger cytotoxic cascades well before extracellular plaque maturation [81]. Together, these studies suggest that intraneuronal pathology arising from lysosomal dysfunction represents a critical stage in AD pathogenesis, offering a mechanistic connection between cellular stress and plaque deposition. More recent mechanistic work underscores the role of autophagy–lysosomal failure (Table 1) in sustaining the amyloidogenic environment. Coffey et al. have shown that alterations in lysosomal pH cause impairment of proteolytic enzyme activity, leading to incomplete degradation of Aβ and tau, further perpetuating aggregate accumulation [82]. A parallel study by Boya and Kroemer extended this understanding by linking lysosomal membrane permeabilization to apoptotic and necrotic cascades, underscoring that lysosomal damage not only fosters amyloid persistence, but also contributes to neuronal loss [83]. These insights converge to suggest that lysosomal dysfunction is not a late byproduct of AD pathology, but a key early defect that initiates a cascade leading to amyloid deposition, tau pathology, and neurodegeneration. By focusing on lysosome-related compartments as therapeutic targets, researchers may be able to intervene upstream of extracellular plaque formation. Approaches aimed at restoring lysosomal pH, stabilizing lysosomal membranes, or enhancing autophagic flux are being actively explored as disease-modifying strategies [84, 85].

However, a major question in the interpretation of autophagic–lysosomal abnormalities in AD is whether the defect reflects a blockage in the fusion of autophagosomes with lysosomes or a failure of degradation within autolysosomes after fusion. Accumulating evidence indicates that the autophagosome–lysosome fusion is not absent but, rather, is highly active in vulnerable neurons. Studies demonstrating robust accumulation of autolysosomes enriched with lysosomal hydrolases such as cathepsin D strongly support that fusion events occur successfully in AD neurons [86–89]. Thus, it is possible that autolysosomes in AD neurons fail to efficiently process their cargoes, leading to the buildup of partially degraded substrates, including lipofuscin granules. As a consequence, the progressive failure of clearance could contribute to the abnormal storage of autophagic material and the expansion of lysosome-derived compartments that are characteristic of AD pathology. Importantly, the presence of lysosomal enzymes in lipofuscin granules [90] further supports the view that disruption of enzymatic activity or lysosomal environment (e.g., pH, membrane integrity, or hydrolase trafficking) results in incomplete substrate degradation. This distinction has significant implications for the interpretation of early pathogenic events. If the primary defect is at the level of fusion, therapeutic strategies would need to focus on promoting autophagosome–lysosome interactions. In contrast, if fusion is intact but degradation fails, then approaches to enhancing lysosomal acidification, stabilizing hydrolase delivery, or stimulating lysosomal clearance are more potential strategies. Recent studies support the latter interpretation by revealing lysosomal acidification defects, cathepsin dysfunction, and impaired lysosomal exocytosis (Table 1) in AD models [47, 70]. Overall, the weight of evidence favors a model in which autophagosomes fuse effectively with lysosomes, but the resulting autolysosomes are incompetent in clearance. This dysfunction could contribute not only to the abnormal accumulation of lipofuscin in neurons, but also to the eventual transformation of degenerating neurons into extracellular plaques containing amyloids and undegraded lysosomal cargoes.

Indeed, Nixon showed that lysosomal impairment drives robust accumulation of lipofuscin-related organelles in AD-affected neurons [47]. This buildup reflects the persistence of undigested membranes, oxidized proteins, and lipids that cannot be cleared due to autophagic flux failure. Importantly, these phenomena are not restricted to late stages; rather, autophagic–lysosomal defects have been observed in preclinical AD models, appearing as some of the earliest cellular abnormalities. This underscores lysosomal dysfunction as a trigger for downstream pathology including amyloid aggregation and neuronal death.

Recent advances have further demonstrated that neurons themselves can harbor Aβ in both soluble and fibrillar forms. Lee and Nixon provided compelling evidence that fibrous Aβ accumulates within degenerating neurons, challenging the view that plaques arise solely from extracellular peptide release. As these compromised neurons undergo degeneration, they release their intracellular amyloid cargo along with lipofuscin and ceroid-containing vesicular organelles into the extracellular space (Fig. 1). This process may directly seed extracellular plaques and account for the abundant cellular debris that persists in plaques long after neuronal loss [70]. Therefore, the autophagic–lysosomal dysfunction not only drives intracellular accumulation of unnecessary material but also determines the fate of neurons as a source of extracellular amyloid plaques.

Furthermore, Cataldo et al. reported that lipofuscin-related organelles and other lysosomal compartments in degenerating neurons are a prominent source of plaque-associated debris in AD, linking neuronal lysosomal dysfunction to extracellular amyloid deposition [65, 91]. These seminal studies established the conceptual foundation for the view that the lysosomal storage materials released from neurons contribute directly to the composition of plaques. Later work by Glabe [81] expanded this concept by demonstrating that neuronal degeneration can drive extracellular amyloid deposition, with amyloid fibrils and lipofuscin-rich organelles persisting as major components of plaques. Pensalfini et al. [92] provided strong evidence that intracellular Aβ aggregates accumulate in vulnerable neurons, and upon neuronal death, are released into the extracellular space where they seed amyloid plaque formation. These findings reinforce that lipofuscin accumulation and amyloidogenic stress within neurons are intimately linked processes that precede plaque emergence. Further evidence has come from studies demonstrating that intraneuronal Aβ accumulation is a critical early event in AD pathogenesis and that neuronal degeneration leads to deposition of this material into the extracellular space, forming the basis of senile plaques [93–95]. These findings highlight that extracellular amyloid plaques are not solely products of extracellular aggregation of soluble Aβ peptides but could also represent the remnants of dying neurons burdened with both amyloid and lipofuscin-related organelles. Most recently, Lee et al. [96] have provided direct in vivo neuropathological evidence from AD mouse models that neurons undergoing lysosomal and autophagic stress accumulate fibrillar Aβ and subsequently degenerate into extracellular plaques, representing a major source of senile plaques. Furthermore, they suggest that these plaques are not simply extracellular accumulations, but a pathological result from neuron-autonomous lysosomal failure, prompting reconsideration of the sequence of events in plaque formation in AD. Together, these converging lines of evidence argue that the release of lipofuscin- and Aβ-containing vesicular debris from degenerating neurons could be a well-supported mechanism of plaque biogenesis.

Moreover, as neurons are metabolically intense and structurally complex postmitotic cells, they accumulate a unique burden of waste products—including lipofuscin—in their lysosomes and residual bodies over time. During aging, their ability to clear damaged macromolecules and organelles decreases. In the presence of injury, oxidative stress, or metabolic collapse, these neurons die, releasing contents including lysosomal debris rich in misfolded proteins and oxidized lipids into the extracellular space to be phagocyted [97–99]. This reconceptualization opens new mechanistic avenues, and raises a question of whether extracellular Aβ is an upstream pathology or merely a consequence.

A critical yet largely overlooked point is that lipofuscin is a potential reservoir for lifelong accumulating bioactive waste, including β-sheet-prone fragments and oxidized membranes [100]. Electron microscopy revealed that lipofuscin granules often contain multilamellar bodies, suggesting partial lysosomal digestion [20, 101]. Spectroscopic analyses revealed protein cross-linking and autofluorescence from advanced glycation and oxidation products [102]. Under conditions of lysosomal alkalinization or oxidative stress, lipofuscin can further impair the autophagic flux, creating a feedback loop of degradative collapse. All these findings indicate that the pigment represents a pre-disease archive, waiting for an opportunity to escape its cytoplasmic confine. Neurons containing high loads of lipofuscin may therefore be stable under homeostasis but primed for extracellular release upon neuronal death.

Neuronal death encompasses diverse mechanisms and depending on the trigger—ischemia, proteotoxicity, mitochondrial dysfunction, or excitotoxicity—can proceed via apoptosis, necroptosis, ferroptosis, or lysosomal rupture [103]. Each pathway leads to a different mode of cellular disassembly, but all share one consequence: the expulsion of intracellular contents into the extracellular space upon death. As such, while much attention has been focused on damage-associated molecular patterns [104] and proinflammatory cytokines released during cell death, another class of molecules—lysosomal waste—has been largely overlooked. In dying neurons, lysosomal membranes become permeable or rupture outright, unleashing their contents into the cytosol and, ultimately, the extracellular milieu.

The released toxic material, which accelerates β-sheet formation in model peptides, including Aβ in vitro [100, 105], cannot be degraded by microglia that are already burdened. Once released into an oxidizing extracellular space, it transits to aggregation-prone peptides. Additionally, inflammatory mediators from nearby glia or peroxidized lipids from neighboring debris may further catalyze oligomerization (Fig. 1). Rather than being a regulated product of APP cleavage within healthy cells, Aβ should be considered a byproduct of incomplete intracellular degradation processes that ultimately accumulate as extracellular deposits. Of note, several studies have raised challenges to the classical Aβ hypothesis [7, 106, 107] and recent evidence suggests that APP plays protective roles in neurodevelopment, synaptic function and disposing nuclear waste through lysosomal exocytosis [108–112].

In summary, defects in lysosomal proteolysis cause incomplete degradation of proteins, abnormal storage of autofluorescent lipopigments such as lipofuscin, and progressive impairment of neuronal function. These changes not only burden neurons metabolically, but also prime them for degeneration. Therefore, neuronal lysosome failure is a critical antecedent to plaque pathology [70, 86].

## Oxidative stress, lipofuscin and tau in neurodegeneration

Oxidative stress plays a pivotal role in the pathogenesis of AD. Elevated levels of ROS have been observed in the brains of affected individuals [113]. The oxidative environment can ultimately cause damage to cellular components, including lipids, proteins, and nucleic acids, causing neuronal demise. Moreover, oxidative modifications of Aβ peptides can increase their aggregation propensity and toxicity, contributing to plaque formation. Oxidative stress and Aβ aggregation may be synergistic. While oxidative stress can promote Aβ aggregation, Aβ can induce oxidative stress by increasing ROS generation, disrupting mitochondrial function, and impairing antioxidant defenses. Oxidative stress can also impair the functions of enzymes involved in Aβ degradation, such as neprilysin and insulin-degrading enzymes, leading to Aβ accumulation [114, 115]. Lipofuscin-induced oxidative stress [53, 116–118] can also impact other cellular processes relevant to AD. For instance, oxidative damage to mitochondrial DNA can impair energy production, leading to neuronal energy deficits and reduced clearance capability [114, 119]. Furthermore, oxidative modifications of the tau protein can promote its hyperphosphorylation and aggregation into NFTs, another hallmark of AD pathology [120]. Thus, lipofuscin-mediated oxidative stress can contribute to multiple facets of AD pathology.

Emerging evidence indicates that lipofuscin is not only associated with Aβ pathology, but also closely linked to tau aggregation and neurodegeneration. Braak et al. [121] have provided a foundational framework for understanding AD pathology, noting that lipofuscin-rich neurons often exhibit vulnerability to early NFT formation. This spatial and temporal overlap suggests that lipofuscin accumulation, as a marker of impaired proteostasis and lysosomal degradation, may facilitate tau hyperphosphorylation and aggregation. Subsequent studies have identified molecular interactions between lipofuscin and proteins implicated in tau pathology. For instance, lipofuscin granules can sequester regulatory proteins such as Pin1, a prolyl isomerase essential for tau dephosphorylation [122]. By binding Pin1, lipofuscin may indirectly enhance tau hyperphosphorylation and destabilize microtubule networks, thereby promoting cytoskeletal disruption. Moreover, the oxidative environment fostered by lipofuscin aggregates could potentiate tau misfolding through modification of cysteine residues and other oxidative stress-sensitive sites on tau [122]. Histopathological analyses have also demonstrated that lipofuscin deposits frequently colocalize with NFTs and dystrophic neurites [123]. In addition, aluminum and other metals enriched within lipofuscin granules, are also found in NFTs. They possibly act as cofactors that accelerate tau fibrillization [66]. In rats, administration of chloroquine (an inhibitor of intralysosomal proteolysis) causes transient accumulation of phosphorylated tau in neuronal lipofuscin granules, particularly in regions such as the amygdala, hippocampus and entorhinal cortex [124]. Furthermore, transmission electron microscopy identified phosphorylated tau and lipofuscin granules in the CA1 region of the hippocampus in mice with noise-induced hearing loss, which also exhibit impairments in both working and recognition memory [125]. These findings underscore the multifaceted effects of lipofuscin on the microenvironment for tau aggregation, which include oxidative damage and metal homeostasis dysregulation.

Together, these studies highlight that lipofuscin should not be viewed solely in the context of amyloid deposition but as a broader driver of neurodegenerative cascades. By impairing proteostasis, sequestering regulatory proteins, and contributing to an environment of oxidative stress, lipofuscin accumulation may actively participate in the initiation and propagation of tau pathology. This reinforces the notion that the age-related pigment is a critical mediator of multiple proteinopathies rather than an inert byproduct of aging.

Therapeutic strategies aimed at reducing the burden of oxidative stress have shown promising results in preclinical models of AD. Antioxidants such as vitamin E, coenzyme Q10, and curcumin have been investigated for their potential to mitigate oxidative damage and improve cognitive function [126–128]. However, clinical trials have yielded mixed results [129–131], highlighting the complexity of oxidative stress in AD and the need for targeted approaches. Targeting lipofuscin and waste accumulation as well as associated oxidative stress may offer a novel avenue for therapeutic intervention. Baldensperger et al. [53] recently developed a method to isolate lipofuscin aggregates from cardiac tissue, and revealed high levels of proline and metals such as calcium and iron in the aggregates. The lipofuscin aggregates were incorporated by fibroblasts and caused pyroptosis-like cell death after incorporation [53, 132]. In addition, lipofuscin accumulation increases mitochondrial ROS and causes lysosomal membrane permeabilization [53]. The interplay between lipofuscin accumulation and oxidative stress in AD provides new insights into therapeutic targets and strategies to mitigate disease progression by restoring lysosomal function and reducing oxidative damage.

## Lipid peroxidation and lipofuscin accumulation

An important contributor to lipofuscin biogenesis is lipid peroxidation, particularly involving polyunsaturated fatty acids (PUFAs). Polyunsaturated acyl chains in cellular membranes are highly susceptible to oxidative attack because of the presence of bis-allylic hydrogen atoms; these hydrogen atoms can be removed by ROS from mitochondrial and lysosomal sources [133]. Once initiated, lipid peroxidation would proceed via free radical chain reactions, producing a variety of secondary products such as malondialdehyde (MDA) and 4-hydroxynonenal (4-HNE). These aldehydes are chemically reactive products that can form covalent adducts with surrounding proteins, lipids, and nucleic acids, resulting in structurally cross-linked, non-degradable complexes that accumulate to form lipofuscin [14]. Lysosomes are the central site of macromolecular turnover; however, peroxidized lipids and aldehydic products are poor substrates for lysosomal hydrolases [134]. As such, instead of being efficiently degraded, these oxidized macromolecules persist within autophagic and endolysosomal compartments, where they undergo further oxidative modification and polymerization, ultimately forming the highly cross-linked aggregates characteristic of lipofuscin [56]. The persistence of such aggregates impairs lysosomal function by occupying degradative capacity and by catalyzing secondary ROS production, further fueling the cycle of lipid peroxidation and pigment deposition [135].

This self-propagating cycle has broad implications for cellular aging and age-associated diseases. Lipid peroxidation and lipofuscin formation are particularly prominent in long-lived post-mitotic cells including neurons, cardiac myocytes, and retinal pigment epithelial cells (RPE), due to the fact that these cells have limited regenerative capacity and undergo ongoing oxidative stress [136]. In these cells, the buildup of lipofuscin not only serves as a marker of cumulative oxidative damage, but also exerts functional consequences, including proteasome inhibition, impaired autophagic flux, and mitochondrial destabilization [136, 137]. Krohne et al. [138] incubated human RPE cells with 4-HNE and MDA components and observed an increase in intracellular accumulation of lipofuscin-like autofluorescence, indicating that lysosomal dysfunction induced by lipid peroxidation increases lipofuscinogenesis and consequently, causes reduced autophagy in RPE cells. Furthermore, H9c2 cardiomyocytes incubated with ferric ammonium citrate and rotenone (which inhibits mitochondrial complex I formation), exhibit higher lipid peroxidation and accumulation of lipofuscin in these cells. Interestingly, the antioxidant SkQ1 and methylene blue (redox mediator) could prevent this lipofuscin accumulation in cardiomyocytes [139]. Thus, lipid peroxidation is not merely a by-product of oxidative stress but a central mechanistic bridge linking ROS production, lysosomal dysfunction, and progressive pigment accumulation.

From a broader pathological perspective, dysregulated lipid peroxidation and lipofuscinogenesis are implicated in neurodegenerative conditions such as AD and Parkinson’s disease, as well as in retinal degeneration like age-related macular degeneration [137]. In this context, excessive peroxidation of membrane PUFAs increases cellular damage by producing cytotoxic aldehydes that ultimately have two outcomes: initiate signaling cascades of cell death, and contribute directly to lipofuscin accumulation. Therapeutic approaches aimed at modulating lipid peroxidation, such as dietary interventions with antioxidants or pharmacological scavengers of lipid radicals, have therefore been explored as strategies to mitigate lipofuscin buildup and its downstream consequences [133, 140, 141].

Another important note is *APOE4*, which remains the highest genetic risk factor for late-onset AD [142]. Recent mechanistic studies have demonstrated that the ApoE4 isoform carrying PUFAs showed an allelic series (ApoE4 > ApoE3 > ApoE2) in inducing lipofuscinosis in human neurons, with profound consequences for lysosomal integrity and neuronal survival [143]. This work establishes a direct link between ApoE isoforms, altered lipid metabolism, and neuronal lipofuscinosis. Importantly, ApoE4 promotes the delivery of PUFA-rich lipid cargoes to neurons, where they are inefficiently processed by the autophagic–lysosomal system. This promotes the accumulation of lipofuscin-related organelles, mirroring the lysosomal pathology observed in vulnerable neuronal populations in AD. The convergence of ApoE-driven lipid metabolism with the failure of lysosomal degradation helps explain the accelerated lysosomal stress in *APOE4* carriers, earlier deposition of lipofuscin in a mouse model of tauopathy, and increased vulnerability of specific neuronal populations.

Beyond amyloid pathology, this *APOE*–lipid–lysosome axis also has implications for tau turnover. The same study [143] showed that alterations in lysosomal lipid composition can cause impairment of the proteolytic pathways for tau degradation. Thus, *APOE4* may also accelerate neuronal lipofuscinosis and disrupt tau proteostasis via lysosomal dysfunction. Together, these findings highlight the importance of considering ApoE isoform in the broader context of lysosomal biology. The findings also strengthen the view that genetic and metabolic risk factors for AD converge on the lysosomal system, driving both amyloid and tau pathology through impaired clearance and lipofuscin buildup.

## Implications for therapeutic strategies

Current AD therapies, such as monoclonal antibodies targeting Aβ (e.g., lecanemab (Leqembi), aducanumab (Aduhelm) from Biogen and Eisai, donanemab (Kisunla) from Lilly, trontinemab (RG6102) from Roche), focus on downstream plaque clearance but have provided variable cognitive outcomes [144–146]. An upstream approach modulating the lysosomal-autophagy pathway and mitigating lipofuscin accumulation, may improve or synergize with existing strategies (Table 2). To achieve this, one major therapeutic implication is to enhance lysosomal biogenesis and function. Although there has been no direct experimental evidence showing that removal of lipofuscin granules leads to clearance of Aβ plaques, or vice versa, enhancing lysosomal function has been demonstrated to reduce both protein aggregates and lipofuscin accumulation. For instance, overexpression of transcription factor EB (TFEB), a master regulator of lysosomal and autophagy genes, reduces both Aβ and tau pathology in mouse models of AD [147, 148]. In addition, targeting TFEB contributes to lipofuscin clearance by upregulating enzymes required for the degradation of oxidized lipids and proteins [149, 150].

**Table 2:** Experimental and therapeutic implications

Methods	Purpose
*Mechanistic elucidation*	
High-resolution proteomics of lipofuscin	Can be conducted to determine whether amyloidogenic peptides are sequestered in lipofuscin bodies from aged neurons. Mass spectrometry of density-gradient-isolated pigment granules could be used to validate the proposed reservoir role of lipofuscin
Immuno-EM of postmortem tissue	Visualization of the colocalization of Aβ and lipofuscin granules can be conducted in situ. Antibody labeling combined with autofluorescence detection could reveal important insights prior to neuronal death
In vitro aggregation assays with lipofuscin extracts	Although challenging, it could assess whether the contents of purified lipofuscin granules accelerate Aβ aggregation. This could inform about seeding capacity and specificity
Single-nucleus sequencing of lipofuscin-burdened cells	Clarification of the transcriptional and functional state of neurons and microglia with high lipofuscin content could inform whether there is a pre-death signature predicting rupture or aggregation susceptibility
*Diagnostic*	
Autofluorescence-based live imaging	It is important to perform tracking of lipofuscin accumulation in transgenic models. Multiphoton or near-infrared probes could help correlate pigment burden with Aβ plaque onset
*Therapeutic interventions*	
Genetic or pharmacologic lipofuscin clearance	Interventions such as TFEB overexpression, mTOR inhibition, or lipid-binding nanoparticles might reduce intracellular pigment and confirm whether this affects downstream amyloid pathology
Senolysis and lysosomal rescue strategies	Navitoclax, dasatinib/quercetin, or lysosomal activators have been used to assess circuit-level effects. It could be useful to explore whether senescent glia or lipofuscin-burdened neurons contribute disproportionately to Aβ deposition

The table describes implications for therapeutic strategies and experimental methods proposed to investigate the relationships between lipofuscin and Aβ

Additionally, strategies that involve chelation of redox-active metals may help neutralize the pro-oxidant activity of lipofuscin. For instance, deferoxamine and clioquinol can effectively limit iron- or copper-mediated oxidative damage in models of neurodegeneration [151–153]. These therapies can simultaneously reduce ROS production and reduce the aggregation of lipofuscin or iron-rich material. Of note, lipofuscin can act as a localized iron reservoir, promoting the Fenton reaction and generation of ROS [154]. This oxidative stress together with lipid peroxidation, constitutes a core trigger of ferroptotic cell death. Furthermore, lipofuscin lysosomal deposition impairs the autophagic flux, including ferritinophagy, which normally regulates iron availability [155]. Disruption of ferritinophagy further increases cytosolic free iron, amplifying lipid peroxidation and ferroptosis susceptibility. These findings highlight the importance of therapies targeting redox-active material.

Another strategy that is receiving interest is the use of compounds that can directly bind, stabilize and remove lipofuscin. For example, β-cyclodextrins, which facilitate lipid clearance and are under investigation in lysosomal storage disorders, may be relevant in reducing lipofuscin buildup [156–158]. β-Cyclodextrins are cyclic oligosaccharides that primarily extract cholesterol from cellular membranes, and have been investigated as therapeutic agents for Niemann-Pick disease type C, a disease associated with cholesterol accumulation in late endosomes and lysosomes [159–161]. In addition to cholesterol extraction, modulation of lipid homeostasis can influence lipofuscin dynamics. For instance, a recent study has demonstrated that activation of liver X receptor by agonists reduces brain lipofuscin accumulation in a primary tauopathy model expressing *APOE4* [143]. Moreover, caloric restriction and fasting mimetics have been shown to increase autophagic flux and delay the age-related accumulation of lipofuscin [162–164], ultimately improving lysosomal function and waste clearance. Nevertheless, therapeutic timing is also an important factor to consider. Lipofuscin accumulation is a slow, progressive process, suggesting that interventions may be most effective if started at preclinical or prodromal stages of disease. Interestingly, a recent study using Death-seq has identified new regulators of senescent cell death [16, 165]. By taking advantage of lipofuscin accumulation in senescent cells, researchers have developed a lipofuscin-binding domain scaffold, which serves as a senolytic platform that can be conjugated with a senolytic drug via an ester bond [16]. Furthermore, improving lysosomal exocytosis [112, 166, 167] may facilitate removal of intracellular waste, thus improving cellular homeostasis.

## Future directions

**Fig. 2:**
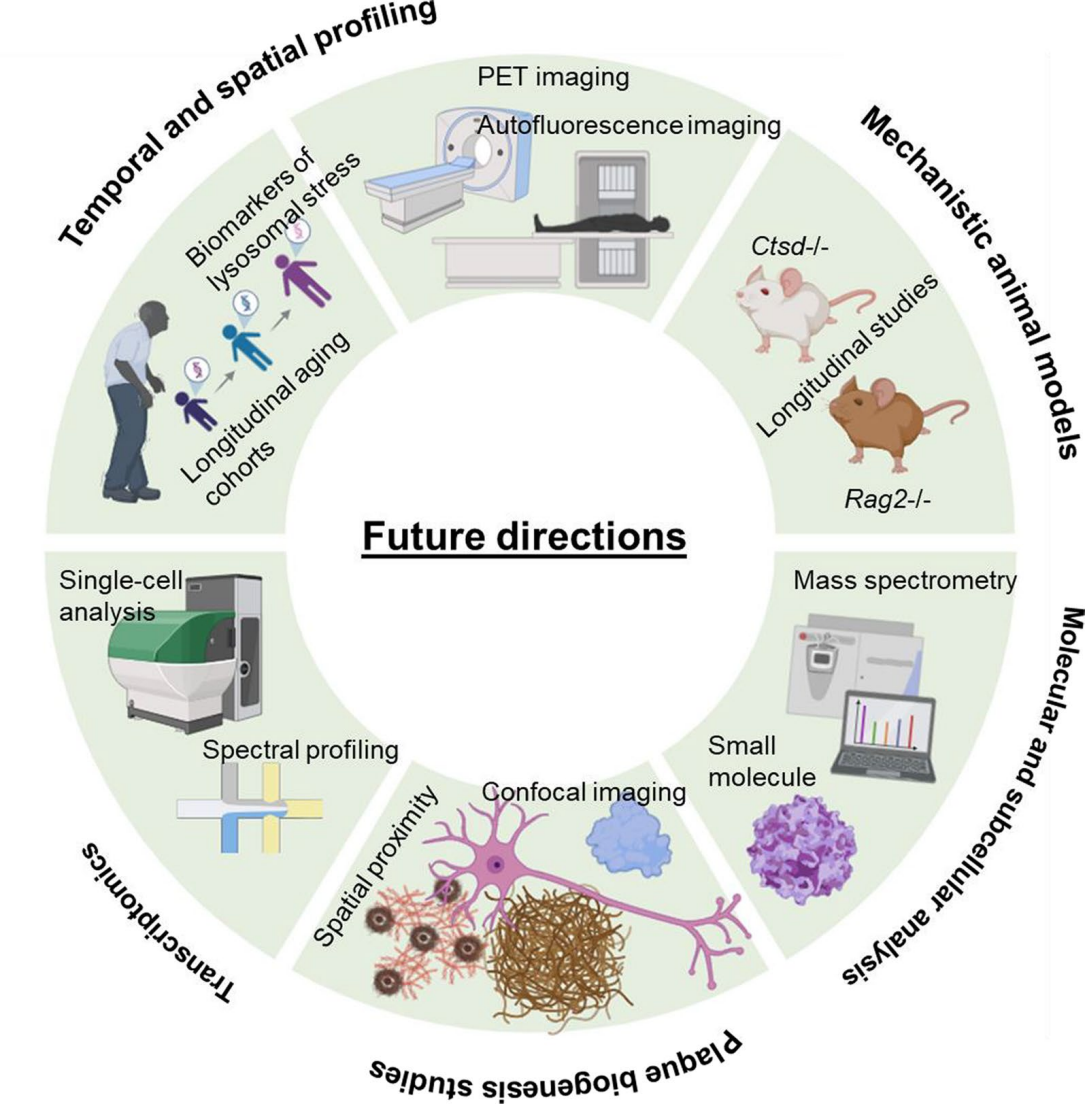
Future research directions for understanding the role of lipofuscin in neurodegeneration. Approaches include longitudinal genetic studies, spatial transcriptomics to map gene expression changes, and subcellular analyses of lipofuscin accumulation. Animal models deficient in genes like *Ctsd*, *Grn*, and *Rag2* exhibit accumulation of lipofuscin and high neurodegenerative pathology, thus can provide mechanistic insights. Lipofuscin-targeted therapies may offer new treatment avenues

While evidence linking lipofuscin to Aβ pathology has been reported, more studies are needed to validate their relationship and develop therapeutically strategies. Therefore, we propose several directions for future research (Fig. 2).

First, more longitudinal studies tracking lipofuscin and Aβ accumulation across aging cohorts are needed. Combining positron emission tomography for Aβ with autofluorescence quantification or novel biomarkers of lysosomal stress could provide insights into the timing of Aβ plaques formation and disease progression. Then, genetic or pharmacological manipulation of lipofuscin accumulation using animal models (such as *Ctsd*^–/–^ and *Rag2*^–/–^ mice that demonstrate rapid lipofuscin accumulation and neurodegeneration) could offer direct evidence of its impact on Aβ pathology [168–170]. However, it is important to highlight that the *Ctsd*^–/–^ mice primarily recapitulate NCL pathology. Other models might be more relevant for studying brain lipofuscin in the context of neurodegeneration, particularly the GRN-linked FTD [171]. By using approaches like brain-penetrant PGRN replacement therapy [172], peripherally administration of AAV expressing brain-penetrant PGRN [173], and AAV9-mediated PGRN expression [174], these models may offer translational implications. Moreover, proteomic and lipidomic mass spectrometry of extracted brain regions in these models could help define the molecular composition of lipofuscin granules and identify shared oxidative signatures or aggregation-prone components [175]. Additionally, subcellular studies using super-resolution microscopy and electron tomography may elucidate whether Aβ and lipofuscin are trafficked through common endolysosomal pathways or whether their spatial proximity is incidental. Also, single-cell and spatial transcriptomic profiling of human postmortem tissue could reveal whether lysosomal and oxidative stress signatures co-segregate with lipofuscin and Aβ burden at the cellular level.

To test whether extracellular lipofuscin actively participates in plaque formation, future studies should combine spectral confocal imaging, autofluorescence quenching, and multiple anti-Aβ/APP-CTF antibodies to rigorously map lipofuscin distribution relative to plaques. For example, correlative ultrastructural analysis and targeted proteomics could clarify whether the released pigment is physically incorporated into plaque matrices. Additionally, longitudinal studies in animal models of neuronal degeneration could reveal whether lipofuscin release precedes or accelerates local Aβ aggregation.

Furthermore, development of lipofuscin-targeted therapies and imaging agents could open translational avenues. This includes small molecules that bind lipofuscin components, enhance lysosomal clearance, or reduce the oxidative load it produces. Additionally, whether lipofuscin directly promotes protein aggregation, or whether it merely reflects failure of clearance, remains an open area of investigation. Future studies should address this point to better understand the roles of lipofuscin in neurodegeneration. Another important point is that lipofuscin may not independently drive protein aggregation but instead represents a biomarker of lysosomal failure that correlates with the buildup of pathogenic proteins. Future research is needed to clarify whether targeting lipofuscin directly, or more broadly, restoring the lysosomal clearance capacity, can alter the trajectory of Aβ and tau accumulation. Genetic or pharmacological interventions that selectively modulate lipofuscin handling could provide key insights into whether lipofuscin is merely a bystander or an active mediator of aggregation pathology in AD.

## Conclusion

Lipofuscin, which has long been considered a passive byproduct of aging, is increasingly being recognized as a dynamic modulator of cellular homeostasis. Lipofuscin accumulation is indicative of lysosomal dysfunction and is closely related to redox imbalance and lipid peroxidation—critical pathways implicated in neurodegenerative diseases, particularly AD. Lipofuscin accumulation may contribute to and exacerbate Aβ accumulation and toxicity by interfering with autophagic clearance and promoting a highly oxidative environment. In this review, we propose a reconsideration of lipofuscin from the “aging marker” or “autofluorescence pigment” to an active player in neurodegeneration and AD pathology. This paradigm shift opens new research directions and therapeutic possibilities. Targeting lipofuscin and its clearance may allow interference of upstream of amyloid plaque formation, preserving proteostasis, reducing oxidative damage, and ultimately slowing or preventing neurodegeneration.

The relationship among aging, lysosomal dysfunction, and neurodegeneration prompts a reconsideration of AD therapies from secretase inhibitors and antibody neutralization toward improving/preserving lysosomal function and preventing neuronal health. Ultimately, we must provide clear answers to not only *how* Aβ forms but also *when*, *from what*, and *why*? Lipofuscin offers a compelling answer to all three questions.

## Data Availability

Not applicable.

## Abbreviations

AD: Alzheimer’s disease
Aβ: Amyloid β
APP: Amyloid precursor protein
APOE: Apolipoprotein E
CTSD: Cathepsin D
GRN: Granulin
4-HNE: 4-Hydroxynonenal
LFB-PAS: Luxol Fast Blue and Periodic Acid-Schiff
MDA: Malondialdehyde
NCL: Neuronal ceroid lipofuscinosis
PGRN: Progranulin
PSEN: Presenilin
PUFAs: Polyunsaturated fatty acids
ROS: Reactive oxygen species
RPE: Retinal pigment epithelial cells
SORL1: Sortilin related receptor 1
TFEB: Transcription factor EB
